# Bayesian evaluation of three serological tests for the diagnosis of bovine brucellosis in Bangladesh

**DOI:** 10.1017/S0950268818003503

**Published:** 2019-01-25

**Authors:** A. K. M. A. Rahman, S. Smit, B. Devleesschauwer, P. Kostoulas, E. Abatih, C. Saegerman, M. Shamsuddin, D. Berkvens, N. K. Dhand, M. P. Ward

**Affiliations:** 1 Department of Medicine, Bangladesh Agricultural University, Mymensingh-2202, Bangladesh; 2 Department of Biomedical Sciences, Institute of Tropical Medicine, Nationalestraat 155, B-2000 Antwerp, Belgium; 3 Research Unit of Epidemiology and Risk Analysis applied to Veterinary Science (UREAR-ULg), Fundamental and Applied Research for Animals & Health (FARAH) Center, Faculty of Veterinary Medicine, University of Liege, Quartier Vallée 2, Avenue de Cureghem 7A, B42, Sart-Tilman Liege, Belgium; 4 Department of Epidemiology and Public Health, Sciensano, Rue Juliette Wytsmanstraat 14, 1050 Brussels, Belgium; 5 Laboratory of Epidemiology, Biostatistics and Animal Health Economics, School of Health Sciences, Faculty of Veterinary Science, University of Thessaly, Karditsa, 224 Trikalon st. 43100, Greece; 6 Department of Applied Mathematics, Computer Science and Statistics, Faculty of Sciences, Ghent University, 281 Krijgslaan, B-9000, Ghent, Belgium; 7 Department of Surgery and Obstetrics, Bangladesh Agricultural University, Mymensingh-2202, Bangladesh; 8 Sydney School of Veterinary Science, The University of Sydney, 425 Werombi Road, Camden, 2570 NSW, Australia

**Keywords:** Animal pathogens, Bayesian analysis, brucellosis, infectious disease epidemiology, veterinary epidemiology

## Abstract

We evaluated the performance of three serological tests – an immunoglobulin G indirect enzyme linked immunosorbent assay (iELISA), a Rose Bengal test and a slow agglutination test (SAT) – for the diagnosis of bovine brucellosis in Bangladesh. Cattle sera (*n* = 1360) sourced from Mymensingh district (MD) and a Government owned dairy farm (GF) were tested in parallel. We used a Bayesian latent class model that adjusted for the conditional dependence among the three tests and assumed constant diagnostic accuracy of the three tests in both populations. The sensitivity and specificity of the three tests varied from 84.6% to 93.7%, respectively. The true prevalences of bovine brucellosis in MD and the GF were 0.6% and 20.4%, respectively. Parallel interpretation of iELISA and SAT yielded the highest negative predictive values: 99.9% in MD and 99.6% in the GF; whereas serial interpretation of both iELISA and SAT produced the highest positive predictive value (PPV): 99.9% in the GF and also high PPV (98.9%) in MD. We recommend the use of both iELISA and SAT together and serial interpretation for culling and parallel interpretation for import decisions. Removal of brucellosis positive cattle will contribute to the control of brucellosis as a public health risk in Bangladesh.

## Introduction

Bovine brucellosis – an economically important reproductive disease of livestock – is one of the most widespread zoonoses globally and remains a major public health problem in many developing countries, including Bangladesh [1]. In humans, person-to-person transmission rarely occurs and disease control primarily depends on the control of brucellosis in animal populations [2]. Human brucellosis cases are geographically clustered in regions with a high animal brucellosis prevalence [3], and control of brucellosis in animals drastically reduces the incidence of human brucellosis [4, 5].

Bovine brucellosis causes infertility, reduced milk yield and calf loss [1]. Both human and animal brucellosis in Bangladesh is caused by *Brucella abortus* [6]. The annual economic loss due to bovine brucellosis in indigenous cows in Bangladesh is estimated to be €720 000, and €12 per cross-bred cow [7]. The reported animal-level seroprevalence in Bangladesh cattle varies from 0% to 18.4% [8], based on the Rose Bengal test (RBT), standard tube agglutination test, interpreted either alone or in series. Since none of the tests used are considered a gold standard, reported prevalence estimates are apparent seroprevalence. Moreover, test performance evaluated in naturally infected Bangladesh cattle did not adjust for imperfect sensitivity (*Se*) and specificity (*Sp*) of the reference test [9].

Prerequisite to brucellosis control/eradication efforts is the correct evaluation of the diagnostic tests that will be used [10] to provide accurate information about disease prevalence. This is needed to estimate disease impact on human health and economic losses, and to design and conduct surveillance programmes. Making policy decisions without knowledge of true disease prevalence can lead to unsuccessful programmes and wastage of limited resources [11].

Diagnostic test performance is traditionally evaluated by comparison to a perfect test (i.e. a gold standard test, assumed to have 100% *Se* and *Sp*). Isolation and identification of *Brucella* spp. is considered to be the ‘gold standard’ for brucellosis diagnosis [12]. However, isolation is difficult to perform in developing countries (lack of trained personnel and sophisticated laboratory facilities with high level safety containment). Bayesian latent class models are increasingly gaining acceptance [13] as a valid alternative for estimating the accuracy of diagnostic tests in the absence of a gold standard. In these models, the true infection status is considered unknown – hence the term latent – and *Se* and *Sp* estimation is based on the cross-classified results of the tests under consideration after their application in multiple populations [14].

In this study, we estimated the *Se* and *Sp* of three serological tests – indirect enzyme linked immunosorbent assay (iELISA), RBT and SAT – using Bayesian latent class models. Further, the animal-level true prevalence of brucellosis in two populations of naturally infected cattle in Bangladesh was estimated.

## Methods

### Study area and animal husbandry practice

**Fig. 1.**
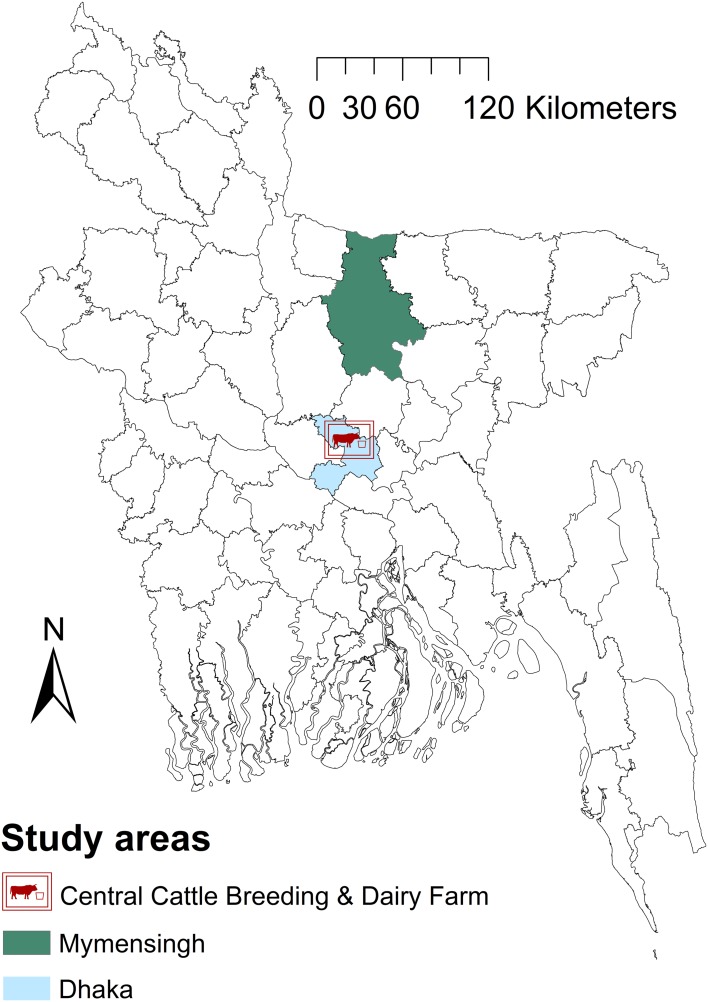
Map of Bangladesh showing the areas included in a study of brucellosis test performance.

The study area was Mymensingh district (MD) and the Government dairy farm (GF) in Savar, located in the Dhaka district of Bangladesh (Fig. 1), located between latitudes 23°31′ and 25°12′N and longitudes 90°01′ and 90°47′E. The areas were chosen because of the location of Bangladesh Agricultural University which manages the brucellosis diagnostic laboratory and because they have the highest livestock population density (>600/km^2^) in Bangladesh.

GF is the largest farm (*n* = 2500) in Bangladesh established to produce crossbred heifers and bulls, collect semen from tested bulls to support the national artificial insemination (AI) programme and to supply milk to Dhaka city. Holstein Friesian and Sahiwal breeds are mainly used for semen production. Cattle management is intensive and only AI is used for reproduction. The cattle management system in MD is small-scale dairy with traditional crop-based subsistence management systems; zero grazing (‘cut-and-carry system’) is mainly practiced, with occasional tethering. The common breeds are indigenous and their crosses with Holstein Friesian and Sahiwal. Both AI and natural service are practiced for reproduction. The study was conducted between September 2007 and August 2008. Vaccination against brucellosis has not been initiated in any livestock species in Bangladesh. The study protocol was approved by the Faculty of Veterinary Science of Bangladesh Agricultural University (01/2007/EB/FVS). Oral consent of farm owners was obtained prior to the collection of blood samples from their cattle. Cattle of MD and the GF were our study populations and cattle of other districts (except dairy intensive regions) and organised farms were our target populations. The response rate of farmers in MD was 100% and the study population and the target population are similar in terms of management.

### Study design and sample size

A cross-sectional study was conducted in the MD of Bangladesh. There is no livestock databank in Bangladesh. A map of MD was digitised (ArcView Version 3.2, Environmental Systems Research Institute, Inc., Redlands, California). Of 146 unions (sub Upa-Zilla, where Upa-Zilla is a subdistrict) in MD, 28 were randomly selected. One geographical coordinate was randomly selected from each selected union and located by a hand-held global positioning system reader. Livestock farmers within 0.5 km of the selected point were informed about the survey [15]. All cattle in a selected farm were sampled. The sample size was calculated using the formula given in equation (1) [16]:1
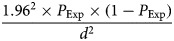

where *P*_Exp_ = expected prevalence = 4.8% (median of previously reported prevalence for the smallholder system) and *d* = precision = 1.5% [17]. These assumptions produce a sample size of 780. As cluster sampling was used, the design effect (*D*) of the study was calculated using the formula [18]:2
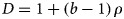
where *b* is the average number of samples per cluster (3) and *ρ* is the intra-cluster correlation coefficient. The intra-cluster correlation coefficient for *B. abortus* infection was reported to be 0.09 [19]. The design effect was therefore calculated to be *D* = 1.2; when multiplied by the calculated sample size, this produced a minimum required sample size of 936. To allow for samples unsuitable for testing, a total of 1020 cattle were sampled.

Blood samples were collected at the GF, including all breeding bulls and a systematic sample of cows (every 10th cow). In addition, a questionnaire designed to collect animal and herd level data was administered during blood sampling of each herd.

### Sample processing and testing

About 5–7 ml of blood was collected from each animal by jugular venipuncture with disposable needles and Venoject tubes, labelled and transported to the laboratory on ice (after clotting) within 12 h of collection. Samples were kept refrigerated (2–8 °C) and the following day sera were separated by centrifuging at 699 ***g*** for 10 min. Sera were stored at −20 °C. Each serum was divided (1–1.5 ml); one aliquot was used for testing and the other was preserved in a serum bank.

All sera were tested in parallel using iELISA, SAT and RBT. These were chosen based on availability, and rapid and easy use within the Bangladesh context. Moreover, simultaneous use of these three tests helps identify acute and chronic cases of brucellosis. The presence of only immunoglobulin G (IgG) indicates chronic brucellosis whereas the presence of both IgG and IgM indicate acute brucellosis [20].

The iELISA was performed according to Limet *et al*. [21] using *B. abortus* biotype 1 (Weybridge 99) S-lipopolysaccharide (*Brucella* smooth lipopolysaccharide) as the antigen. A detailed description of the method can be found in Rahman *et al*. [22]. The cut-off value for a positive result was set to 5 IU/ml [23] of test serum in MD and 12.5 IU/ml in GF [24] in the Bangladesh context. The RBT was performed as described by Alton *et al*. [12]. The procedure has been described in detail in a previous paper by Rahman *et al*. [22]. The result was considered positive when agglutination was noted after 4 min. The SAT was carried out with ethylene diamine tetra acetic acid as described by Garin *et al*. [25]. Reading was performed on the basis of degree of agglutination and expressed in international units (IU). Any serum with an antibody titre greater than or equal to 30 IU/ml, as prescribed by the EU [26], was considered positive. Those performing the tests were blinded to the results of the other tests.

### Bayesian latent class analysis

A Bayesian analysis framework was used in OpenBUGS [27] and R 3.3.1 (The R Foundation for Statistical Computing, Vienna, Austria) to estimate the diagnostic accuracy of the three tests and the true prevalence of infection in the two subpopulations. An important consideration in the evaluation of multiple diagnostic tests is the assumption of conditional independence between tests, given the true disease status. Two tests are considered to be conditionally independent if the sensitivity or specificity of one test does not depend on the results of the other test.

As fully explained in Berkvens *et al*. [28], converting apparent prevalence into true prevalence requires one to solve a system of over-parameterised equations. This invariably requires the input of external (prior) information, either in the form of prior estimates of test sensitivity or test specificity, or in the form of some hypothesis such as conditional independence of tests or constancy of test characteristics across different populations. Several solutions have been proposed, from the Hui and Walter [29] model based on two conditionally independent diagnostic tests applied in two populations with sensitivity and specificity constant over the two populations, to the fully parameterised models proposed by e.g. [28, 30]. We used an extension of the conditional dependence model described by Gardner *et al*. [30] for three tests considering constancy of test characteristics in two populations.

### Definition of infection status

Bayesian latent class models create their own probabilistic definition of infection that depends on what analyte the tests actually detect (e.g. organisms or immune responses to organisms). This definition of the infection status must be interpreted and communicated from a biological perspective [31].

In this study, all tests detect the humoral response of the host: iELISA detects only IgG antibodies, whereas RBT detects mainly IgG antibodies (though also detects IgM and IgA) and SAT detects mainly IgM (but also detects IgG and IgA). Hence, the latent infection under consideration is unambiguous and defined as the presence and persistence of *Brucella* within an animal long enough to produce a detectable humoral immune response at any time during their life.

### Modelling conditional dependence

Let *pr*_1_ and *pr*_2_ be the true prevalence in MD and the GF respectively, and *T*_1_,*T*_2_ and *T*_3_ represent the test outcomes for iELISA, RBT and SAT, respectively, with positive test outcomes represented by 1 (or +) and negative test outcomes by 0 (or −). Sensitivities and specificities are denoted by *Se* and *Sp*. The covariances between iELISA and RBT, between iELISA and SAT, between RBT and SAT and among iELISA, RBT and SAT in infected cattle are denoted by *a*_12_, *a*_13_, *a*_23_, *a*_123_ and in non-infected cattle by *b*_12_, *b*_13_, *b*_23_, *b*_123_, respectively. The probability of an animal from the first population (MD) testing positive in three tests is given in equation (3):3
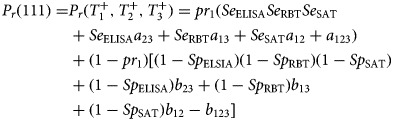


The multinomial cell probabilities for the other combinations in the first population – as well as in the second population (GF) – are calculated in a similar manner (see Supplementary file 1 for details).

The upper and lower limits of the sensitivity covariance between iELISA and RBT (i.e. *a*_12_) are derived from the following probabilities given in equation (4):4
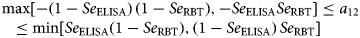


The other covariances between two tests and among three tests are calculated in a similar manner (see Supplementary file 1 for details).

To minimise the dependency of the model estimates on the specified prior information we adopted a semi-dependent model [32] that reduced the number of parameters to be estimated. Specifically, the covariances of the tests in non-infected cattle (i.e. the dependencies between the *Sp*s of the tests) were forced to be zero. A trade off exists between modelling accurately all dependencies, which leads to loss of identifiability, and the number of parameters to be estimated within the model. Hence, it is recommended to drop dependence terms with minimal impact on the final estimates [31] so as to reduce the dependency of the model estimates on prior information. Based on the existing literature, the *Sp*s of the tests were expected to be high and close to unity [33]. Hence, omitting the dependencies among *Sp*s of highly specific tests has a minimal impact on the estimated parameters. Moreover, the covariances among the tests in infected cattle (i.e. the dependencies between the *Se*s of the tests) were forced to be positive to capture the biologically plausible assumption that tests based on the same biological principle have positively rather than negatively correlated results. The OpenBUGS model code used to estimate true prevalence and characteristics of the diagnostic tests is provided in Supplementary file 1.

### Prior information on test characteristics

The external (prior) information was generated through meta-analysis by the ‘metandi’ [34] command in Stata 13.1 (Statistical Software: Release 13, College Station, TX, USA) using the results published in the following references: iELISA [33, 35–39]; RBT [33, 35, 38, 40–42] and SAT [33, 35, 43, 44]. A brief description of the meta-analysis based on the cited references is given in Supplementary file 3.

*β* distributions were used as priors for the parameters of interest (*Se*s, *Sp*s and prevalences). A uniform prior distribution in the range 0–1 was chosen for both prevalences and sensitivity of SAT, *β*(1, 1). *β* distributions for the priors on *Se*s and *Sp*s of three tests (Table 1) were calculated using the ‘betaExpert’ function of the package ‘prevalence’ [45] in R 3.3.1.

**Table 1.** Summary values of the meta-analysis estimation of characteristics and corresponding *β* distribution parameters for three serological tests for detection of *Brucella* antibodies

	Sensitivity		Specificity		Sensitivity		Specificity	
Test	Mean	95% CI	Mean	95% CI	*α*	*β*	*α*	*β*
iELISA	93.9	86.9–97.2	99.8	99.1–99.9	85.00	6.4867	750.00	2.6544
RBT	91.0	70.6–97.7	99.6	84.3–99.9	18.80	2.7583	22.60	1.0837
SAT	82.6	27.8–98.3	99.7	97.4–99.9	3.45	1.5142	190.00	1.6483

CI, confidence interval.

Prior information on the four covariance parameters (for infected cattle) were not available and were generated in R 3.3.1 based on the range of possible values of the sensitivities and specificities listed in Table 1 (see Supplementary file 1).

### Sensitivity analysis

To assess the influence of prior information [13] on the estimates of the model parameters three additional, different sets of prior information were considered: (a) uniform priors in the range 0 to 1 for *Se*s and *Sp*s (i.e. *β*(1,1) distribution); (b) uniform priors in the range 0–1 for *Sp*s and the informative priors for *Se*s used for the primary analysis and (c) uniform priors in the range 0–1 for *Se*s and the informative priors for *Sp*s used for the primary analysis.

### Model implementation

The model was run with a burn-in period of 50 000 iterations and estimates were based on a further 50 000 iterations using three chains. Model selection proceeded according to the method described in Berkvens *et al*. [28], making use of Deviance Information Criterion (DIC), pD and Bayes-*p*. Moreover, the convergence of the model was also assessed by time-series plots, Gelman Rubin convergence diagnostics, autocorrelation plots and Monte Carlo standard errors [46].

### Adherence to the STARD-BLCM guidelines

For this study, we followed the STARD-BLCM reporting guidelines on the design, conduct and results of diagnostic accuracy studies (see Supplementary file 2) that use Bayesian latent class models [31].

## Results

### Descriptive statistics

#### Mymensingh district (MD)

A total of 1020 cattle were subjected to the three serological tests in MD. Most (86% and 70%) of the cattle in MD were indigenous and female, respectively. The median age was 3 years (interquartile range (IQR), 1.5 to 6 years). The median body weight of cattle was 90.0 kg (IQR 50.0 to 120.0 kg). The herd size ranged from 1 to 11 with a median of two animals. Most (74%) of the herds consisted of only one, two or three cattle. Only one herd had more than 10 cattle. The average herd level apparent prevalence of brucellosis observed in the MD of Bangladesh was 2.48% (9/362).

#### Government dairy farm (GF)

In the GF, 340 sera samples (including 89 from breeding bulls) were tested by three serological tests (Table 2). The median age of cattle was 4 years (IQR 3 to 8 years). Most (88% and 64%) of the GF cattle were cross-bred and female, respectively. The median body weight of cattle was 200 kg (IQR 152 to 353 kg).

**Table 2.** Cross-classified test results for brucellosis in cattle in Mymensingh district (MD) and Government dairy farm (GF) of Bangladesh

Serological test			Location		Usual interpretation of test result
iELISA	RBT	SAT	MD	GF	
1	1	1	1	44	Acute infection
1	1	0	1	2	Acute infection
1	0	1	2	5	Acute infection
1	0	0	5	2	Chronic infection
0	1	1	1	15	Acute infection
0	1	0	6	1	
0	0	1	9	3	
0	0	0	995	268	
		Total	1020	340	

iELISA, indirect enzyme-linked immunosorbent assay; RBT, Rose Bengal test; SAT, slow agglutination test.

### Serological results

Table 2 shows the numbers of animals that tested positive in the three tests. Only 6.1% (22/362) of herds from MD were serologically positive to at least one of the three tests (one animal and two animals positive per herd in 19 and 3 herds, respectively). Only 0.49% (5/1020) of cattle were both acutely and chronically infected in MD, and about 2.5% (25/1020) of cattle were positive in at least one serological test. The apparent prevalences were 0.9% (9/1020) based on iELISA and RBT, and 1.3% (13/1020) based on SAT.

In the GF, 19.4% (66/340) of cattle were acutely infected with brucellosis and only 0.6% (2/340) of cattle were chronically infected. About 21.2% (72/340) of cattle were positive in at least one serological test in the GF. The apparent prevalence was 15.6% (53/340), 18.2% (62/340) and 19.7% (67/340) based on iELISA, RBT and SAT, respectively.

### Meta-analysis

Table 1 summarises the results of the meta-analysis and the corresponding parameters for the respective prior *β* distributions.

### Characteristics of diagnostic tests and true prevalence

The posterior estimates of true prevalence, sensitivity, specificity and sensitivity covariances of diagnostic tests are provided in Table 3. The true estimated prevalence of brucellosis among cattle in MD and the GF were 0.6% (95% confidence interval (CI) 0.2–1.2) and 20.4% (95% CI 16.2–24.8), respectively. The covariance between RBT and SAT – in infected cattle – was 14.3%. The positive predictive value (PPV) and negative predictive value (NPV) of iELISA, RBT and SAT for the diagnosis of bovine brucellosis in Bangladesh are presented in Table 4. Table 5 presents the sensitivity, specificity, PPV, NPV and performance index of serial (an animal is considered positive if it is positive in all tests) and parallel (an animal is considered positive if it is positive in at least one test) interpretation of test combinations for the diagnosis of bovine brucellosis in Bangladesh. Serial interpretation of iELISA and SAT and RBT and SAT yielded the highest PPV (99.9%) in the GF. The highest PPV (99.2%) was observed for serial interpretation of iELISA and RBT in MD. Whereas, the parallel interpretation of iELISA and SAT, iELISA and RBT and RBT and SAT in MD yielded very high NPVs (99.9%). In GF, the parallel interpretation of iELISA and SAT produced the highest NPV (99.6%). The performance indices varied from 1.75 to 1.97.

**Table 3.** Posterior estimates of prevalence (%) of brucellosis, sensitivity, specificity and sensitivity covariances of iELISA, RBT and SAT at MD and GF, Bangladesh

Variable	Mean	95% credibility interval
Prevalence (MD)	0.6	0.2–1.2
Prevalence (GF)	20.4	16.2–24.8
Sensitivity: iELISA	84.6	78.6–89.9
Specificity: iELISA	99.6	99.2–99.8
Sensitivity: RBT	87.4	79.4–93.7
Specificity: RBT	99.4	98.9–99.8
Sensitivity: SAT	93.7	86.4–98.4
Specificity: SAT	99.2	98.6–99.6
Covariance between tests		
iELISA and RBT in infected cattle (*a*_12_)	6.8	0.2–22.5
RBT and SAT in infected cattle (*a*_23_)	14.3	0.5–40.9
iELISA and SAT in infected cattle (*a*_13_)	6.9	0.2–23.1

**Table 4.** Positive and negative predictive values of iELISA, RBT and SAT for the diagnosis of bovine brucellosis at MD and GF, Bangladesh

	MD		GF	
Variable	Mean	95% CrI	Mean	95% CrI
Prevalence	0.6	0.2–1.2	20.4	16.2–24.8
PPV: iELISA	51.8	22.5–78.2	97.9	96.2–99.2
NPV: iELISA	99.9	99.8–1	96.2	94.3–97.7
PPV: RBT	45.2	18.1–72.8	97.3	94.9–98.9
NPV: RBT	99.9	99.8–1	96.8	94.6–98.5
PPV: SAT	38.7	15.1–64.2	96.5	93.9–98.4
NPV: SAT	99.9	99.9–1	98.4	96.4–99.6

PPV, positive predictive value; NPV, negative predictive value; MD, Mymensingh district; GF, Government farm; CrI, credibility interval.

**Table 5:** Sensitivity, specificity, PPV, NPV and performance index of serial and parallel interpretation of test combinations for the diagnosis of bovine brucellosis in Bangladesh

Parameter	MD		GF		Sensitivity	Specificity	PI
	PPV (95% CrIs)	NPV (95% CrIs)	PPV (95% CrIs)	NPV (95% CIs)	Mean (95% CrIs)	Mean (95% CrIs)	Mean (95% CrIs)
Parallel between iELISA and RBT	34.7 (12.9–58.7)	99.9 (99.9–100)	99.9 (99.9–100)	99.3 (98.5–99.7)	97.2 (94.4–98.9)	98.9 (98.4–99.4)	1.96 (1.93–1.98)
Serial between iELISA and RBT	99.2 (97.3–99.9)	99.9 (99.8–100)	99.3 (98.0–99.9)	94.3 (91.6–96.5)	74.4 (66.5–82.1)	100 (99.9–100)	1.75 (1.67–1.82)
Parallel between RBT and SAT	28.0 (10.0–49.4)	99.9 (99.9–100)	94.4 (91.3–96.9)	99.5 (98.4–99.9)	97.9 (93.9–99.7)	98.6 (97.8–99.1)	1.97 (1.93–1.98)
Serial between RBT and SAT	98.7 (95.7–99.7)	99.9 (99.8–100)	99.9 (99.9–100)	96.3 (93.6–98.3)	83.1 (73.9–90.6)	99.9 (99.9–100)	1.83 (1.74–1.91)
Parallel between iELISA and SAT	30.5 (11.0–52.9)	99.9 (99.9–100)	95.1 (92.2–97.3)	99.6 (98.9–99.9)	98.4 (96.1–99.6)	98.7 (98.1–99.2)	1.97 (1.95–1.99)
Serial between iELISA and SAT	98.9 (96.7–99.8)	99.9 (99.8–100)	99.9 (99.9–100)	96.3 (93.6–98.3)	79.8 (71.9–86.6)	100 (99.9–100)	1.80 (1.72–1.87)

PI, performance index (sensitivity + specificity).

### Sensitivity analysis

The results of the sensitivity analysis are presented in Table 6. The posterior specificity estimates for the three tests and sensitivity estimates of RBT and SAT were similar. As expected the influence of specified prior information on the posterior estimates for the sensitivity of the iELISA was minor (Tables 3 and 6).

**Table 6.** *Se* and *Sp* estimates under alternative prior specifications (sensitivity analysis)

	Sensitivity (*Se*)	Specificity (*Sp*)
Models and tests	Mean (95% CrIs)	Mean (95% CrIs)
Uniform priors in the range 0 to 1 for *Se*s and *Sp*s (i.e. *β*(1,1) distribution)		
ELISA	72.2 (59.3–82.7)	99.0 (98.3–99.6)
RBT	87.6 (73.9–96.8)	99.2 (98.5–99.6)
SAT	93.3 (80.4–99.5)	98.7 (97.9–99.3)
Uniform priors in the range 0 to 1 for *Sp*s and the informative priors for *Se*s used for the primary analysis		
ELISA	85.4 (79.3–90.8)	99.1 (98.4–99.6)
RBT	89.4 (80.9–95.9)	99.1 (98.4–99.6)
SAT	95.1 (87.2–99.6)	98.6 (97.8–99.3)
Uniform priors in the range 0 to 1 for *Se*s and the informative priors for *Sp*s used for the primary analysis		
ELISA	72.3 (58.7–82.9)	99.4 (98.9–99.7)
RBT	86.1 (71.2–95.3)	99.2 (98.7–99.7)
SAT	92.6 (77.6–99.2)	98.9 (98.3–99.4)

## Discussion

We estimated the performance of three serological tests for the diagnosis of bovine brucellosis in Bangladesh. Such information is needed by clinicians and decision-makers in the context of clinical diagnoses or quantitative risk assessments, as well as for prevalence estimation or risk factor studies [12]. This information is also critical for designing control programmes to reduce brucellosis in cattle and indirectly in humans as a public health risk.

Serial interpretation of the iELISA-SAT produced the highest PPV in GF and high PPV in MD (i.e. decreased false positive results). A higher probability of being diseased given a test positive result is helpful for culling decisions. In contrast, parallel interpretation of iELISA and SAT yielded highest NPV in both MD and the GF (i.e. minimised false negative results). A higher probability of being healthy given a test negative result is helpful in import decisions [13]. Based on study results and brucellosis biology (discussed below), we recommend the combined use of IgG iELISA and SAT and their serial or parallel interpretation depending on the intended use: culling or import decisions, respectively.

The benefit of using SAT is that it can detect IgM antibody. Simultaneous presence of IgM and IgG indicates acute brucellosis; IgG alone is an indication of chronic brucellosis [20]. The simultaneous use (one serum is tested by both tests) and serial interpretation of SAT and IgG iELISA helps define the stage of brucellosis in animals. We found that 0.49% and 19.4% cattle were acutely infected with brucellosis in MD and the GF, respectively. Culling of these acutely infected cattle will reduce the spread of infection in cattle populations and thereby risk of brucellosis in humans.

We estimated the true prevalence of brucellosis applying multiple tests in parallel on blood samples from cattle in Bangladesh. This is an essential piece of information for decision makers before implementing prevention and control measures. We estimated – via a Bayesian analysis framework – the prevalence of brucellosis in cattle in MD and the GF to be 0.6% (95% CI 0.2–1.2) and 20.4% (95% CI 16.2–24.8), respectively. Estimated true prevalence in MD is even lower than the lower limit of previous apparent prevalence estimates, which ranged from 1.1% to 10.6% [8]. The smaller sample size, non-randomness of sample collection and types and interpretation of tests used may be responsible for the great variation in prevalences estimated in previous studies (e.g. 9.7% estimated by Ahasan *et al*. [9]).

In Bangladesh, indigenous cattle are reared in a subsistence management system whereas in commercial management systems mostly cross-bred cattle are maintained. The prevalence of brucellosis is reported to be significantly higher in the commercial production system [8]. This is also supported by our data: most (86%) cattle in this study (in MD) are indigenous breed, reflecting the general breed distribution in Bangladesh. Farmers are aware of the disease and cows showing signs suggestive of brucellosis are usually sold to butchers. Moreover, around 3.5 million cattle are slaughtered annually in Bangladesh, about 40% during the festival of Eid-ul-Azha [47]. During this mass slaughter, the carcasses of animals infected with brucellosis may be removed from the population, partially explaining the very low prevalence in subsistence management systems such as in MD. As a result of the shorter life span of animals, there is a lower risk of *Brucella* transmission in this population because this disease is most common in sexually mature animals [48, 49]. In addition, the small size of the herds in MD might also be responsible for lower prevalence of brucellosis [50]. In such a low prevalence situation, test and slaughter policies to control brucellosis can be successfully implemented [51].

The prevalence in the GF exceeds the upper limit of the previous prevalence reports. There might be several reasons, including larger herd size, irregularly/not testing cattle, high proportion of cross-breed cattle, recent cattle introductions without proper testing and the sole use of AI at the GF. AI using semen from brucellosis infected bulls can spread disease [6, 52].

In Bangladesh the main source of human brucellosis is occupational, rather than foodborne [2]. Recommendations to use vaccines against brucellosis in Bangladesh for the first time in herds in which the prevalence is very high (as in the largest GF) should be made with caution. Vaccines such as S19 can interfere with serological diagnosis, can induce abortion if administered during pregnancy and S19 strain also infects humans [53]. The RB51 vaccine does not induce antibody responses that are detected by conventional brucellosis serologic tests. However, this vaccine can induce abortion, infects humans [53] and this vaccine strain is resistant to rifampicin, a widely used antibiotic in the treatment of human brucellosis [54]. Therefore, we do not recommend the introduction of vaccine for brucellosis in Bangladesh at this time. More studies are needed to determine the status (and prevalence) of brucellosis in commercial dairy farms.

The model we used is non-identifiable because the degrees of freedom in the data are fewer than the parameters to be estimated. Hence some of our estimates depend on the specified priors; a fact that was also indicated from the change in the posterior estimates (especially the *Se* of iELISA) under alternative priors. That is why – as is the case with any non-identifiable model – the sound justification for the selection of priors is imperative [31].

One study limitation might be that findings do not represent the brucellosis status in dairy intensive regions of Bangladesh. We have assumed that the prevalence in farms of MD is similar because the cattle herd size and management practice in MD are similar. Brucellosis is endemic in MD, and currently there is no control programme in place. Thus, MD farms are expected to fall within the same prevalence ‘window’ and the distribution of the infection stages between these farms is expected to be similar. Another issue is the size of the sample we have chosen for Bayesian analysis. There is no unique way to estimate sample size requirements under Bayesian analysis and especially for these types of models. Most methods adopt a case-specific stochastic simulation approach and required sample sizes also depend on the existing information that will be incorporated as priors. Hence, it is often an iterative approach that cannot be specified a priori. Standard formulas for prevalence estimation – as the one used here – generally produce larger sample sizes and are more transparent for the broader audience. Hence, their use here but in other similar papers.

In Bangladesh, bovine brucellosis in small-scale dairy and subsistence management systems appears to be controlled naturally without any directed control measures. Simultaneous use and serial and parallel interpretation of iELISA and SAT help culling and animal importation decisions, respectively. Surveillance in conjunction with a test-and-cull approach will reduce the prevalence of brucellosis in commercial dairy farms even in a resource poor setting. Controlling brucellosis in animals and increasing awareness of risk factors for human brucellosis might also reduce the level of exposure and thereby the disease.

## Supplementary material

For supplementary material accompanying this paper visit https://doi.org/10.1017/S0950268818003503. click here to view supplementary material

## References

[1] FAO (2006) WHO, Brucellosis in humans and animals. Food and Agriculture Organization. Available at http://www.who.int/csr/resources/publications/Brucellosis.pdf (Accessed October 2017).

[2] Seleem MN, Boyle SM and Sriranganathan N (2010) Brucellosis: a re-emerging zoonosis. Veterinary Microbiology 140, 392–398. 19604656 10.1016/j.vetmic.2009.06.021

[3] De Massis F (2005) Correlation between animal and human brucellosis in Italy during the period 1997–2002. Clinical Microbiology and Infection 11, 632–636. 16008615 10.1111/j.1469-0691.2005.01204.x

[4] Zinsstag J (2007) Human benefits of animal interventions for zoonosis control. Emerging Infectious Diseases 13, 527–531. 17553265 10.3201/eid1304.060381 PMC2725951

[5] Pappas G (2006) The new global map of human brucellosis. The Lancet Infectious Diseases 6, 91–99. 16439329 10.1016/S1473-3099(06)70382-6

[6] Rahman AKMA (2017) *Brucella abortus* is prevalent in both humans and animals in Bangladesh. Zoonoses and Public Health 64, 394–399. 28068003 10.1111/zph.12344

[7] Islam A (1983) Economic losses due to brucellosis among cattle in Bangladesh. Bangladesh Veterinary Journal 17, 57–62.

[8] Islam MA (2013) A review of *Brucella* seroprevalence among humans and animals in Bangladesh with special emphasis on epidemiology, risk factors and control opportunities. Veterinary Microbiology 166, 317–326. 23867082 10.1016/j.vetmic.2013.06.014

[9] Ahasan MS (2016) Bovine and caprine brucellosis in Bangladesh: Bayesian evaluation of four serological tests, true prevalence, and associated risk factors in household animals. Tropical Animal Health and Production 49, 1–11. 27628065 10.1007/s11250-016-1151-1

[10] Greiner M and Gardner I (2000) Epidemiologic issues in the validation of veterinary diagnostic tests. Preventive Veterinary Medicine 45, 3–22. 10802331 10.1016/s0167-5877(00)00114-8

[11] McDermott J, Grace D and Zinsstag J (2013) Economics of brucellosis impact and control in low-income countries. Revue Scientifique Et Technique 32, 249–261. 23837382 10.20506/rst.32.1.2197

[12] Alton G (1988) Techniques for the Brucellosis Laboratory. Paris, France: INRA, pp. 112–189.

[13] Arif S (2018) Evaluation of three serological tests for diagnosis of bovine brucellosis in smallholder farms in Pakistan by estimating sensitivity and specificity using Bayesian latent class analysis. Preventive Veterinary Medicine 149, 21–28. 29290297 10.1016/j.prevetmed.2017.11.002

[14] Black MA and Craig BA (2002) Estimating disease prevalence in the absence of a gold standard. Statistics in Medicine 21, 2653–2669. 12228883 10.1002/sim.1178

[15] Cringoli G (2002) A cross-sectional coprological survey of liver flukes in cattle and sheep from an area of the southern Italian Apennines. Veterinary Parasitology 108, 137–143. 12208041 10.1016/s0304-4017(02)00183-8

[16] Dohoo I, Martin W and Stryhn H (2009) Veterinary Epidemiologic Research. Charlottetown, Canada: AVC Inc.

[17] Pourhoseingholi MA, Vahedi M and Rahimzadeh M (2013) Sample size calculation in medical studies. Gastroenterology and Hepatology from Bed to Bench 6, 14–17. 24834239 PMC4017493

[18] Bennett S (1991) A simplified general method for cluster-sample surveys of health in developing countries. World Health Statistics Quarterly 44, 98–106. 1949887

[19] Otte MJ and Gumm ID (1997) Intra-cluster correlation coefficients of twenty infections calculated from the results of cluster-sample surveys. Preventive Veterinary Medicine 31, 147–150. 9234433 10.1016/s0167-5877(96)01108-7

[20] Godfroid J, Nielsen K and Saegerman C (2010) Diagnosis of brucellosis in livestock and wildlife. Croatian Medical Journal 51, 296–230. 20718082 10.3325/cmj.2010.51.296 PMC2931434

[21] Limet J (1988) Le diagnostic serologique de la brucellose bovine par ELISA. Annaes De Medecine Veterinaire 132, 565–575.

[22] Rahman AKMA (2012) Seroprevalence and risk factors for brucellosis in a high-risk group of individuals in Bangladesh. Foodborne Pathogens and Disease 9, 190–197. 22300225 10.1089/fpd.2011.1029

[23] Rahman AKMA (2015) Epidemiology of brucellosis in humans and domestic ruminants in Bangladesh (PhD thesis). 20 Boulevard de Colonster, The University of Liege, Liege, Belgium, 189 pp.

[24] Smit S (2013) Bovine brucellosis in Bangladesh: Estimation of true prevalence and diagnostic test-characteristics (Master thesis). Faculty of Bioscience Engineering, Ghent University, Belgium.

[25] Garin B, Trap D and Gaumont R (1985) Assessment of the EDTA seroagglutination test for the diagnosis of bovine brucellosis. Veterinary Record 117, 444–445. 3934838 10.1136/vr.117.17.444

[26] Shey-Njila O (2005) Serological survey of bovine brucellosis in Cameroon. The Revue d’élevage et Médecine Vétérinaire des Pays Tropicaux 58, 139–143.

[27] Spiegelhalter D (2007) OpenBUGS user manual, version 3.0. 2. MRC Biostatistics Unit, Cambridge.

[28] Berkvens D (2006) Estimating disease prevalence in a Bayesian framework using probabilistic constraints. Epidemiology 17, 145–153. 16477254 10.1097/01.ede.0000198422.64801.8d

[29] Hui SL and Walter SD (1980) Estimating the error rates of diagnostic tests. Biometrics 36, 167–171. 7370371

[30] Gardner IA (2000) Conditional dependence between tests affects the diagnosis and surveillance of animal diseases. Preventive Veterinary Medicine 45, 107–122. 10802336 10.1016/s0167-5877(00)00119-7

[31] Kostoulas P (2017) STARD-BLCM: standards for the reporting of diagnostic accuracy studies that use Bayesian Latent Class Models. Preventive Veterinary Medicine 138, 37–47. 28237234 10.1016/j.prevetmed.2017.01.006

[32] Kostoulas P (2006) Application of a semi-dependent latent model in the Bayesian estimation of the sensitivity and specificity of two faecal culture methods for diagnosis of paratuberculosis in sub-clinically infected Greek dairy sheep and goats. Preventive Veterinary Medicine 76(1-2), 121–134. 16797753 10.1016/j.prevetmed.2006.04.008

[33] Abernethy D (2012) Field trial of six serological tests for bovine brucellosis. The Veterinary Journal 191, 364–370. 21550272 10.1016/j.tvjl.2011.03.008

[34] Harbord RM and Whiting P (2009) Metandi: meta-analysis of diagnostic accuracy using hierarchical logistic regression. Stata Journal 9, 211.

[35] Van Aert A (1984) A comparative study of ELISA and other methods for the detection of *Brucella* antibodies in bovine sera. Veterinary Microbiology 10, 13–21. 6442027 10.1016/0378-1135(84)90052-x

[36] Dohoo I (1986) A comparison of five serological tests for bovine brucellosis. Canadian Journal of Veterinary Research 50, 485–493. 3539295 PMC1255253

[37] Uzal FA (1995) Evaluation of an indirect ELISA for the diagnosis of bovine brucellosis. Journal of Veterinary Diagnostic Investigation 7, 473–475. 8580167 10.1177/104063879500700408

[38] Samartino L (1999) Validation of enzyme-linked immunosorbent assays for the diagnosis of bovine brucellosis. Veterinary Microbiology 70, 193–200. 10596803 10.1016/s0378-1135(99)00122-4

[39] Saegerman C (2004) Evaluation of three serum i-ELISAs using monoclonal antibodies and protein G as peroxidase conjugate for the diagnosis of bovine brucellosis. Veterinary Microbiology 100, 91–105. 15135517 10.1016/j.vetmic.2004.02.003

[40] Dajer A (1999) Evaluation of a fluorescence-polarization assay for the diagnosis of bovine brucellosis in Mexico. Preventive Veterinary Medicine 40, 67–73. 10343334 10.1016/s0167-5877(99)00004-5

[41] Mainar-Jaime RC (2005) Specificity dependence between serological tests for diagnosing bovine brucellosis in *Brucella*-free farms showing false positive serological reactions due to *Yersinia enterocolitica* O: 9. Canadian Veterinary Journal 46, 913–916. 16454384 PMC1255594

[42] Muma JB (2007) Risk factors for brucellosis in indigenous cattle reared in livestock-wildlife interface areas of Zambia. Preventive Veterinary Medicine 80, 306–317. 17481753 10.1016/j.prevetmed.2007.03.003

[43] Stemshorn B (1985) A comparison of standard serological tests for the diagnosis of bovine brucellosis in Canada. Canadian Journal of Comparative Medicine 49, 391–394. 4075239 PMC1236197

[44] Lord V, Rolo M and Cherwonogrodzky J (1989) Evaluation of humoral immunity to *Brucella* sp. in cattle by use of an agar-gel immunodiffusion test containing a polysaccharide antigen. American Journal of Veterinary Research 50, 1813–1816. 2515779

[45] Devleesschauwer B (2015) Package ‘prevalence’: Tools for prevalence assessment studies, R package version 0.4.0.

[46] Gelman A and Rubin DB (1992) Inference from iterative simulation using multiple sequences. Statistical Science 7, 457–472.

[47] Anon (2007) National livestock development policy. Ministry of Fisheries and Livestock, Government of the People's Republic of Bangladesh. Available at http://www.dls.gov.bd/files/LivestockPolicyFinal.pdf (Accessed October 2017).

[48] Lopes L, Nicolino R and Haddad J (2010) Brucellosis- risk factors and prevalence: a review. The Open Veterinary Science Journal 4, 72–84.

[49] Godfroid J (2004) Infectious Diseases of Livestock. UK, Oxford University Press.

[50] Al-Majali AM (2009) Seroprevalence and risk factors for bovine brucellosis in Jordan. Journal of Veterinary Science 10, 61–65. 19255525 10.4142/jvs.2009.10.1.61 PMC2801095

[51] Hegazy Y, Ridler A and Guitian F (2009) Assessment and simulation of the implementation of brucellosis control programme in an endemic area of the Middle East. Epidemiology and Infection 137, 1436–1448. 19288957 10.1017/S0950268809002301

[52] Chiebao DP (2013) Variables Associated with Infections of Cattle by *Brucella abortus*, *Leptospira* spp. *and Neospora spp.* in Amazon Region in Brazil. Transboundary and Emerging Diseases 62, e30–e36. 26302373 10.1111/tbed.12201

[53] Olsen S and Tatum F (2010) Bovine brucellosis. The Veterinary Clinics of North America. Food Animal Practice 26, 15–27. 20117540 10.1016/j.cvfa.2009.10.006

[54] Ariza J (2007) Perspectives for the treatment of brucellosis in the 21st century: the Ioannina recommendations. PLoS Medicine 27, e317. 10.1371/journal.pmed.0040317 PMC2222927 18162038

