# Supplementary material for: Bayesian evaluation of three serological tests for the diagnosis of bovine brucellosis in Bangladesh
Source: Epidemiol Infect. 2019 Jan 25;147:e73. doi: 10.1017/S0950268818003503 (PMC6518595; doi:10.1017/S0950268818003503)
Supplement: Supplementary file 1 [file S0950268818003503sup001.zip › S1_OpenBUGS_code.rtf]

# Bayesian code accompanying the following manuscript:# Rahman et al (2018) Bayesian evaluation of three serological tests  # for the diagnosis of bovine brucellosis in Bangladesh.######################### Variable descriptions ######################################### Diagnostic tests: # Indirect IgG ELISA (iELISA)# Rose Bengal Test (RBT)# Serum Agglutination Test (SAT)#### Prevalences: # pr1: Prevalence of brucellosis in population 1, i.e. Mymensingh district (MD)# pr2: Prevalence of brucellosis in population 2, i.e. a government farm (GF)#### Sensitivities (se) and specificities (sp):# se: Sensitivities of diagnostic tests # se[1]: se of iELISA ; # se[2]: se of RBT ;# se[3]: se of SAT ;# sp: Specificities of diagnostic tests;# sp[1]: sp of iELISA ; # sp[2]: sp of RBT ;# sp[3]: sp of SAT ;#### Sensitivity dependence# a12: Sensitivity dependence between iELISA and RBT # a13: Sensitivity dependence between iELISA and SAT # a23: Sensitivity dependence between RBT and SAT # a123: Sensitivity dependence among iELISA, RBT and SAT #### Specificity dependences# b12: Specificity dependence between iELISA and RBT # b13: Specificity dependence between iELISA and SAT # b23: Specificity dependence between RBT and SAT# b123: Specificity dependence among iELISA, RBT and SAT # Data and population #r1: Number of positive cattle in MD#r2: Number of positive cattle in GF#n1: Number of cattle in MD#n2: Number of cattle in GF#p1: Multinomial cell probability in MD#p2: Multinomial cell probability in GF######################### Model #####################################model{# Multinomial distributionsr1[1:8] ~ dmulti(p1[1:8], n1)r2[1:8] ~ dmulti(p2[1:8], n2)#### Probabilities of tests to be positive in the first population (MD)# p(1 1 1): Probability of all three tests to be positive in MD p1[1]<-(pr1)*(se[1]*se[2]*se[3] + se[1]*a23 + se[2]*a13+ se[3]*a12 + a123) + (1-pr1)*((1-sp[1])*(1-sp[2])*(1-sp[3]) + (1-sp[1])*b23 + (1-sp[2])*b13 + (1-sp[3])*b12 - b123)# p(1 1 0): Probability of first two test to be positive and the third test to be negative in MD p1[2] <- pr1*(se[1]*se[2]*(1-se[3]) - se[1]*a23 - se[2]*a13 + (1-se[3])*a12 - a123) + (1-pr1)*((1-sp[1])*(1-sp[2])*sp[3] - (1-sp[1])*b23 - (1-sp[2])*b13 + sp[3]*b12 + b123)# p(1 0 1): Probability of first and third test to be positive but the second test negative in MDp1[3] <- pr1*(se[1]*(1-se[2])*se[3] - se[1]*a23 + (1-se[2])*a13 - se[3]*a12-a123) + (1-pr1)*((1-sp[1])*sp[2]*(1-sp[3]) - (1-sp[1])*b23+sp[2]*b13 - (1-sp[3])*b12 + b123)# p(1 0 0):Probability of first test to be positive but the second and third tests negative in MDp1[4] <- pr1*(se[1]*(1-se[2])*(1-se[3]) + se[1]*a23 - (1-se[2])*a13 - (1-se[3])*a12+a123) + (1-pr1)*((1-sp[1])*sp[2]*sp[3] + (1-sp[1])*b23 - sp[2]*b13 - sp[3]*b12 - b123)# p(0 1 1):Probability of first test to be negative but the second and third tests positive in MDp1[5] <- pr1*((1-se[1])*se[2]*se[3] + (1-se[1])*a23 - se[2]*a13 - se[3]*a12-a123) + (1-pr1)*(sp[1]*(1-sp[2])*(1-sp[3]) + sp[1]*b23 - (1-sp[2])*b13 - (1-sp[3])*b12 + b123)# p(0 1 0):Probability of first and third test to be negative but the second test positive in MDp1[6] <- pr1*((1-se[1])*se[2]*(1-se[3]) - (1-se[1])*a23 + se[2]*a13 - (1-se[3])*a12+a123) + (1-pr1)*(sp[1]*(1-sp[2])*sp[3] - sp[1]*b23 + (1-sp[2])*b13 - sp[3]*b12 - b123)#p (0 0 1): Probability of first two test to be negative and the third test to be positive in MD p1[7] <- pr1*((1-se[1])*(1-se[2])*se[3] - (1-se[1])*a23 - (1-se[2])*a13 + se[3]*a12 + a123) + (1-pr1)*(sp[1]*sp[2]*(1-sp[3]) - sp[1]*b23 - sp[2]*b13 + (1-sp[3])*b12 - b123)#p( 0 0 0): Probability of all three tests to be negative in MDp1[8] <- pr1*((1-se[1])*(1-se[2])*(1-se[3]) + (1-se[1])*a23 + (1-se[2])*a13 + (1-se[3])*a12-a123) + (1-pr1)*(sp[1]*sp[2]*sp[3] + sp[1]*b23 + sp[2]*b13 + sp[3]*b12 + b123)#### Probabilities of tests to be positive in the second population (GF)# # p(1 1 1): Probability of all three tests to be positive in GFp2[1] <- pr2*(se[1]*se[2]*se[3] + se[1]*a23 + se[2]*a13 + se[3]*a12+a123) + (1-pr2)*((1-sp[1])*(1-sp[2])*(1-sp[3]) + (1-sp[1])*b23 + (1-sp[2])*b13 + (1-sp[3])*b12 - b123)# p(1 1 0): Probability of first two test to be positive and the third test to be negative in GF p2[2] <- pr2*(se[1]*se[2]*(1-se[3]) - se[1]*a23 - se[2]*a13 + (1-se[3])*a12-a123) + (1-pr2)*((1-sp[1])*(1-sp[2])*sp[3] - (1-sp[1])*b23 - (1-sp[2])*b13 + sp[3]*b12 + b123)# p(1 0 1): Probability of first and third test to be positive but the second test negative in GFp2[3] <- pr2*(se[1]*(1-se[2])*se[3] - se[1]*a23 + (1-se[2])*a13 - se[3]*a12 - a123) + (1-pr2)*((1-sp[1])*sp[2]*(1-sp[3]) - (1-sp[1])*b23 + sp[2]*b13 - (1-sp[3])*b12 + b123)# p(1 0 0):Probability of first test to be positive but the second and third tests negative in GFp2[4] <- pr2*(se[1]*(1-se[2])*(1-se[3]) + se[1]*a23 - (1-se[2])*a13 - (1-se[3])*a12+a123) + (1-pr2)*((1-sp[1])*sp[2]*sp[3] + (1-sp[1])*b23 - sp[2]*b13 - sp[3]*b12 - b123)# p(0 1 1):Probability of first test to be negative but the second and third tests positive in GFp2[5] <- pr2*((1-se[1])*se[2]*se[3] + (1-se[1])*a23 - se[2]*a13 - se[3]*a12-a123) + (1-pr2)*(sp[1]*(1-sp[2])*(1-sp[3]) + sp[1]*b23 - (1-sp[2])*b13 - (1-sp[3])*b12 + b123)# p(0 1 0):Probability of first and third test to be negative but the second test positive in GFp2[6] <- pr2*((1-se[1])*se[2]*(1-se[3]) - (1-se[1])*a23 + se[2]*a13 - (1-se[3])*a12+a123) + (1-pr2)*(sp[1]*(1-sp[2])*sp[3] - sp[1]*b23 + (1-sp[2])*b13 - sp[3]*b12 - b123)#p (0 0 1): Probability of first two test to be negative and the third test to be positive in GF p2[7] <- pr2*((1-se[1])*(1-se[2])*se[3] - (1-se[1])*a23 - (1-se[2])*a13 + se[3]*a12+a123) + (1-pr2)*(sp[1]*sp[2]*(1-sp[3]) - sp[1]*b23 - sp[2]*b13 + (1-sp[3])*b12 - b123)#p( 0 0 0): Probability of all three tests to be negative in GFp2[8] <- pr2*((1-se[1])*(1-se[2])*(1-se[3]) + (1-se[1])*a23 + (1-se[2])*a13 + (1-se[3])*a12 - a123) + (1-pr2)*(sp[1]*sp[2]*sp[3] + sp[1]*b23 + sp[2]*b13 + sp[3]*b12 + b123)#### Prior information for se and sp of three testspr1 ~ dbeta(1,1) # Prevalence of brucellosis in population 1 (MD)pr2 ~ dbeta(1,1)# Prevalence of brucellosis in population 2 (GF)se[1] ~dbeta(85.0,6.4867) # Se: 93.9se[2] ~ dbeta(18.8,2.7583) # Se: 91.0se[3] ~ dbeta(1,1)sp[1] ~ dbeta(750.00,2.6544) # Sp: 99.8sp[2] ~ dbeta(22.6,1.0837) # Sp: 99.6sp[3] ~ dbeta(190.00,1.6483) # Sp: 99.7#### Lower and upper limits and distribution of covariances between sensitivities of...# ...iELISA and RBT ll1 <- max(-(1-se[1])*(1-se[2]), -se[1]*se[2])ul1 <- min(se[1]*(1-se[2]),(1-se[1])*se[2])a12 ~ dunif(0,ul1)# ...iELISA and SAT ll2 <- max(-(1-se[1])*(1-se[3]), -se[1]*se[3])ul2 <- min(se[1]*(1-se[3]),(1-se[1])*se[3])a13 ~ dunif(0,ul2)# ...RBT and SAT in GFll3 <- max(-(1-se[2])*(1-se[3]), -se[2]*se[3])ul3 <- min(se[2]*(1-se[3]),(1-se[2])*se[3])a23 ~ dunif(0,ul3)#### Lower and upper limits and distribution of covariances between specificities of...# ...iELISA and RBT ll4 <- max(-(1-sp[1])*(1-sp[2]), -sp[1]*sp[2])ul4 <- min(sp[1]*(1-sp[2]),(1-sp[1])*sp[2])b12 <-0# ...iELISA and SAT ll5 <- max(-(1-sp[1])*(1-sp[3]), -sp[1]*sp[3])ul5 <- min(sp[1]*(1-sp[3]),(1-sp[1])*sp[3])b13  <-0# ...RBT and SAT ll6 <- max(-(1-sp[2])*(1-sp[3]), -sp[2]*sp[3])ul6 <- min(sp[2]*(1-sp[3]),(1-sp[2])*sp[3])b23  <-0#### Lower and upper limits and distribution of covariances among sensitivities of...# ...all three tests together ll71 <- -(se[1]*se[2]*se[3]+se[1]*a23+se[2]*a13+se[3]*a12)ll72 <- -((1-se[1])*(1-se[2])*se[3]-(1-se[1])*a23-(1-se[2])*a13+se[3]*a12)ll73 <- -((1-se[1])*se[2]*(1-se[3])-(1-se[1])*a23+se[2]*a13-(1-se[3])*a12)ll74 <- -(se[1]*(1-se[2])*(1-se[3])+se[1]*a23-(1-se[2])*a13-(1-se[3])*a12)ll7 <- max(max(ll71, ll72), max(ll73, ll74))ul71 <- (1-se[1])*se[2]*se[3]+(1-se[1])*a23-se[2]*a13-se[3]*a12ul72 <- se[1]*(1-se[2])*se[3]-se[1]*a23+(1-se[2])*a13-se[3]*a12ul73 <- se[1]*se[2]*(1-se[3])-se[1]*a23-se[2]*a13+(1-se[3])*a12ul74 <- (1-se[1])*(1-se[2])*(1-se[3])+(1-se[1])*a23+(1-se[2])*a13+(1-se[3])*a12ul7 <- min(min(ul71, ul72), min(ul73, ul74))l1l <- min(ll7,ul7)u1l <- max(ll7,ul7)a123 ~ dunif(0,u1l)#### Lower and upper limits and distribution of covariances among specificities of...# ... all three tests together ll81 <- -(sp[1]*sp[2]*sp[3]+sp[1]*b23+sp[2]*b13+sp[3]*b12)ll82 <- -((1-sp[1])*(1-sp[2])*sp[3]-(1-sp[1])*b23-(1-sp[2])*b13+sp[3]*b12)ll83 <- -((1-sp[1])*sp[2]*(1-sp[3])-(1-sp[1])*b23+sp[2]*b13-(1-sp[3])*b12)ll84 <- -(sp[1]*(1-sp[2])*(1-sp[3])+sp[1]*b23-(1-sp[2])*b13-(1-sp[3])*b12)ll8<- max(max(ll81, ll82), max(ll83, ll84))ul81 <- (1-sp[1])*sp[2]*sp[3]+(1-sp[1])*b23-sp[2]*b13-sp[3]*b12ul82 <- sp[1]*(1-sp[2])*sp[3]-sp[1]*b23+(1-sp[2])*b13-sp[3]*b12ul83 <- sp[1]*sp[2]*(1-sp[3])-sp[1]*b23-sp[2]*b13+(1-sp[3])*b12ul84 <- (1-sp[1])*(1-sp[2])*(1-sp[3])+(1-sp[1])*b23+(1-sp[2])*b13+(1-sp[3])*b12ul8<- min(min(ul81, ul82), min(ul83, ul84))l2l<- min(ll8,ul8)u2l <- max(ll8,ul8)b123 <-0#Estimation of the posterior predictive p-value [bayesp1 (MD) and bayesp2 (GF)] for ( i in 1:8){d1[i] <- r1[i]*log(max(r1[i],1)/(p1[i]*n1))}G0 <- 2*sum(d1[])r11[1:8] ~ dmulti(p1[1:8],n1)for (i in 1:8){d11[i] <- r11[i]*log(max(r11[i],1)/(p1[i]*n1))}Gt <- 2*sum(d11[])bayesp1 <- step(G0 - Gt)for ( i in 1:8){d2[i] <- r2[i]*log(max(r2[i],1)/(p2[i]*n2))}G10 <- 2*sum(d2[])r22[1:8] ~ dmulti(p2[1:8],n2)for (i in 1:8){d22[i] <- r22[i]*log(max(r22[i],1)/(p2[i]*n2))}G1t <- 2*sum(d22[])bayesp2 <- step(G10 - G1t)#### Covariances between test pairs   # (a in infected and b in noninfected cattle)# Between sensitivities of...# ...iELISA and RBT cova12 <- a12/(sqrt(se[1]*(1-se[1]))*sqrt(se[2]*(1-se[2])))# ...RBT and SATcova23 <- a23/(sqrt(se[2]*(1-se[2]))*sqrt(se[3]*(1-se[3])))# ...iELISA and SATcova13 <- a13/(sqrt(se[1]*(1-se[1]))*sqrt(se[3]*(1-se[3])))# Between specificities of...# ...iELISA and RBTcovb12 <- b12/(sqrt(sp[1]*(1-sp[1]))*(sp[2]*(1-sp[2])))# ...RBT and SATcovb23 <- b23/(sqrt(sp[2]*(1-sp[2]))*(sp[3]*(1-sp[3])))# ...iELISA and SATcovb13 <- b13/(sqrt(sp[1]*(1-sp[1]))*(sp[3]*(1-sp[3])))#Positive Predictive Value (PPV) of  iELISA in MDppvelMD<- (se[1]*pr1)/( se[1]*pr1+(1-sp[1])*(1-pr1))#PPV of RBT in MDppvrbMD<-(se[2]*pr1)/ (se[2]*pr1+(1-sp[2])*(1-pr1))#PPV of SAT in MDppvsatMD<-(se[3]*pr1)/( se[3]*pr1+(1-sp[3])*(1-pr1))#Negative Predictive Value (NPV) of iELISA in MDnpvelMD<- (sp[1]*(1-pr1))/( (1-se[1])*pr1+sp[1]*(1-pr1))#NPV of RBT in MDnpvrbMD<-(sp[2]*(1-pr1))/ ((1-se[2])*pr1+sp[2]*(1-pr1))#NPV of SAT in MDnpvsatMD<-(sp[3]*(1-pr1))/ ((1-se[3])*pr1+sp[3]*(1-pr1))#PPV of iELISA in GFppvelGF<-(se[1]*pr2)/(se[1]*pr2+(1-sp[1])*(1-pr2))#PPV of RBT in GFppvrbGF<-( se[2]*pr2)/( se[2]*pr2+(1-sp[2])*(1-pr2))#PPV of SAT in GFppvsatGF<- (se[3]*pr2)/( se[3]*pr2+(1-sp[3])*(1-pr2))#NPV of iELISA GFnpvelGF<- (sp[1]*(1-pr2))/((1-se[1])*pr2+sp[1]*(1-pr2))#NPV of RBT in GFnpvrbGF<-( sp[2]*(1-pr2)) /((1-se[2])*pr2+sp[2]*(1-pr2))#NPV of SAT in GFnpvsatGF<-(sp[3]*(1-pr2))/ ((1-se[3])*pr2+sp[3]*(1-pr2))# Se, Sp, PI, PPV, NPV using all three tests simultaneously# Parallel combination of iELISA,RBPT, SAT: Classify + if any test +#Specificity_parallel_iELISA and RBTsp.par.12 <- sp[1]*sp[2]+b12#Sensitivity parallel_iLEISA and RBTse.par.12 <- 1-((1-se[1])*(1-se[2])+a12)#Specificity_parallel_iELISA and SATsp.par.13 <- sp[1]*sp[3]+b13#Sensitivity parallel_iLEISA and SATse.par.13 <- 1-((1-se[1])*(1-se[3])+a13)#Specificity_parallel_RBT and SATsp.par.23 <- sp[2]*sp[3]+b23#Sensitivity parallel_RBT and SATse.par.23 <- 1-((1-se[2])*(1-se[3])+a23)#Performance Index_parallelpi.par.12 <- se.par.12 + sp.par.12pi.par.13 <- se.par.13 + sp.par.13pi.par.23 <- se.par.23 + sp.par.23# Serial combination of iELISA, RBT, SAT: Classify + if all tests +#Sensitivity_serial_iELISA ans RBTse.ser.12 <- se[1]*se[2]+a12#Specificity_serial_iELISA ans RBTsp.ser.12 <- 1-((1-sp[1])*(1-sp[2])+b12)#Sensitivity_serial_iELISA ans SATse.ser.13 <- se[1]*se[3]+a13#Specificity_serial_iELISA ans SATsp.ser.13 <- 1-((1-sp[1])*(1-sp[3])+b13)#Sensitivity_serial_RBT ans SATse.ser.23 <- se[2]*se[3]+a23#Specificity_serial_RBT ans RBTsp.ser.23 <- 1-((1-sp[2])*(1-sp[3])+b23)#Performance Index_serialpi.ser.12 <- se.ser.12 + sp.ser.12pi.ser.13 <- se.ser.13 + sp.ser.13pi.ser.23 <- se.ser.23 + sp.ser.23#Positive and negative predictive values_parallel_serial_Mymensingh districtppv.par.MD.12 <- (pr1*se.par.12)/(pr1*se.par.12+(1-pr1)*(1-sp.par.12))ppv.ser.MD.12<- (pr1*se.ser.12)/(pr1*se.ser.12+(1-pr1)*(1-sp.ser.12))npv.par.MD.12 <- (1-pr1)*sp.par.12/((1-pr1)*sp.par.12+pr1*(1-se.par.12))npv.ser.MD.12 <- (1-pr1)*sp.ser.12/((1-pr1)*sp.ser.12+pr1*(1-se.ser.12))ppv.par.MD.13 <- (pr1*se.par.13)/(pr1*se.par.13+(1-pr1)*(1-sp.par.13))ppv.ser.MD.13 <- (pr1*se.ser.13)/(pr1*se.ser.13+(1-pr1)*(1-sp.ser.13))npv.par.MD.13 <- (1-pr1)*sp.par.13/((1-pr1)*sp.par.13+pr1*(1-se.par.13))npv.ser.MD.13 <- (1-pr1)*sp.ser.13/((1-pr1)*sp.ser.13+pr1*(1-se.ser.13))ppv.par.MD.23 <- (pr1*se.par.23)/(pr1*se.par.23+(1-pr1)*(1-sp.par.23))ppv.ser.MD.23 <- (pr1*se.ser.23)/(pr1*se.ser.23+(1-pr1)*(1-sp.ser.23))npv.par.MD.23 <- (1-pr1)*sp.par.23/((1-pr1)*sp.par.23+pr1*(1-se.par.23))npv.ser.MD.23 <- (1-pr1)*sp.ser.23/((1-pr1)*sp.ser.23+pr1*(1-se.ser.23))#Positive and negative predictive values_parallel_serial_Government farmppv.par.GF.12 <- (pr2*se.par.12)/(pr2*se.par.12+(1-pr2)*(1-sp.par.12))ppv.ser.GF.12<- (pr2*se.ser.12)/(pr2*se.ser.12+(1-pr2)*(1-sp.ser.12))npv.par.GF.12 <- (1-pr2)*sp.par.12/((1-pr2)*sp.par.12+pr2*(1-se.par.12))npv.ser.GF.12 <- (1-pr2)*sp.ser.12/((1-pr2)*sp.ser.12+pr2*(1-se.ser.12))ppv.par.GF.13 <- (pr2*se.par.13)/(pr2*se.par.13+(1-pr2)*(1-sp.par.13))ppv.ser.GF.13 <- (pr2*se.ser.13)/(pr2*se.ser.13+(1-pr2)*(1-sp.ser.13))npv.par.GF.13 <- (1-pr2)*sp.par.13/((1-pr2)*sp.par.13+pr2*(1-se.par.13))npv.ser.GF.13 <- (1-pr2)*sp.ser.13/((1-pr2)*sp.ser.13+pr2*(1-se.ser.13))ppv.par.GF.23 <- (pr2*se.par.23)/(pr2*se.par.23+(1-pr2)*(1-sp.par.23))ppv.ser.GF.23 <- (pr2*se.ser.23)/(pr2*se.ser.23+(1-pr2)*(1-sp.ser.23))npv.par.GF.23 <- (1-pr2)*sp.par.23/((1-pr2)*sp.par.23+pr2*(1-se.par.23))npv.ser.GF.23 <- (1-pr2)*sp.ser.23/((1-pr2)*sp.ser.23+pr2*(1-se.ser.23))}######################### Data #####################################list(r1=c(1,1,2,5,1,6,9,995),n1=1020,r2=c(44,2,5,2,15,1,3,268), n2=340)#initial values for covariances generated in R (code attached below)list(pr1=0.5, se=c( 0.93,0.77,0.22),sp=c(0.95,0.99,0.7),a12= 0.0189 ,a13= 0.0196 ,a23= 0.0594,a123= 0.001134,r11=c(1,1,2,5,1,6,9,995))list(pr1=0.5,se=c(0.965,0.835,0.6),sp=c(0.975,0.995,0.85),a12=0.011725,a13=0.0035 ,a23= 0.0165,a123=0.000595000000000001,r11=c(1,1,2,5,1,6,9,995))list(pr1=0.5,se=c(0.98,0.9,0.98),sp=c(0.999,0.999,0.999),a12=0.00800000000000001,a13=0.00960000000000001,a23=0.00800000000000001,a123= 0.00368,r11=c(1,1,2,5,1,6,9,995))##R code to generate initial values for covariances###1pr =0.5; se=c(0.93,0.77,0.22) ; sp= c(0.95,0.99,0.70);p=array (0, 8)ll1 = max(-(1-se[1])*(1-se[2]), -se[1]*se[2]); ul1 = min(se[1]*(1-se[2]),(1-se[1])*se[2])ll2 = max(-(1-se[1])*(1-se[3]), -se[1]*se[3]); ul2 = min(se[1]*(1-se[3]),(1-se[1])*se[3])ll3 = max(-(1-se[2])*(1-se[3]), -se[2]*se[3]); ul3 = min(se[2]*(1-se[3]),(1-se[2])*se[3])a12 = mean(c(ll1,ul1)); a13 = mean(c(ll2,ul2)); a23 = mean(c(ll3,ul3))ll4 = max(-(1-sp[1])*(1-sp[2]), -sp[1]*sp[2]); ul4 = min(sp[1]*(1-sp[2]),(1-sp[1])*sp[2])ll5 = max(-(1-sp[1])*(1-sp[3]), -sp[1]*sp[3]); ul5 = min(sp[1]*(1-sp[3]),(1-sp[1])*sp[3])ll6 = max(-(1-sp[2])*(1-sp[3]), -sp[2]*sp[3]); ul6 = min(sp[2]*(1-sp[3]),(1-sp[2])*sp[3])b12 = mean(c(ll4,ul4)); b13 = mean(c(ll5,ul5)); b23 = mean(c(ll6,ul6))ll71 = -(se[1]*se[2]*se[3]+se[1]*a23+se[2]*a13+se[3]*a12)ll72 = -((1-se[1])*(1-se[2])*se[3]-(1-se[1])*a23-(1-se[2])*a13+se[3]*a12)ll73 = -((1-se[1])*se[2]*(1-se[3])-(1-se[1])*a23+se[2]*a13-(1-se[3])*a12)ll74 = -(se[1]*(1-se[2])*(1-se[3])+se[1]*a23-(1-se[2])*a13-(1-se[3])*a12)ll7 = max(ll71, ll72, ll73, ll74)ul71 = (1-se[1])*se[2]*se[3]+(1-se[1])*a23-se[2]*a13-se[3]*a12ul72 = se[1]*(1-se[2])*se[3]-se[1]*a23+(1-se[2])*a13-se[3]*a12ul73 = se[1]*se[2]*(1-se[3])-se[1]*a23-se[2]*a13+(1-se[3])*a12ul74 = (1-se[1])*(1-se[2])*(1-se[3])+(1-se[1])*a23+(1-se[2])*a13+(1-se[3])*a12ul7 = min(ul71, ul72, ul73, ul74)a123 = mean(c(ll7,ul7))ll81 = -(sp[1]*sp[2]*sp[3]+sp[1]*b23+sp[2]*b13+sp[3]*b12)ll82 = -((1-sp[1])*(1-sp[2])*sp[3]-(1-sp[1])*b23-(1-sp[2])*b13+sp[3]*b12)ll83 = -((1-sp[1])*sp[2]*(1-sp[3])-(1-sp[1])*b23+sp[2]*b13-(1-sp[3])*b12)ll84 = -(sp[1]*(1-sp[2])*(1-sp[3])+sp[1]*b23-(1-sp[2])*b13-(1-sp[3])*b12)ll8 = max(ll81, ll82, ll83, ll84)ul81 = (1-sp[1])*sp[2]*sp[3]+(1-sp[1])*b23-sp[2]*b13-sp[3]*b12ul82 = sp[1]*(1-sp[2])*sp[3]-sp[1]*b23+(1-sp[2])*b13-sp[3]*b12ul83 = sp[1]*sp[2]*(1-sp[3])-sp[1]*b23-sp[2]*b13+(1-sp[3])*b12ul84 = (1-sp[1])*(1-sp[2])*(1-sp[3])+(1-sp[1])*b23+(1-sp[2])*b13+(1-sp[3])*b12ul8 = min(ul81, ul82, ul83, ul84)b123 = mean(c(ll8,ul8))print(paste("list(pr=0.5, se=c(", se[1], ",", se[2], ",", se[3], "), sp=c(", sp[1], ",", sp[2], ",", sp[3], "), a12=", a12, ",a13=", a13, ",a23=", a23, ",b12=", b12, ",b13=", b13, ",b23=", b23, ",a123=", a123, ",b123=", b123, ", r2=c(1,1,2,5,1,6,9,995))"), quote=F)###2pr =0.5; se=c(0.965,0.835,0.6) ; sp= c(0.975,0.995,0.85);p=array (0, 8)ll1 = max(-(1-se[1])*(1-se[2]), -se[1]*se[2]); ul1 = min(se[1]*(1-se[2]),(1-se[1])*se[2])ll2 = max(-(1-se[1])*(1-se[3]), -se[1]*se[3]); ul2 = min(se[1]*(1-se[3]),(1-se[1])*se[3])ll3 = max(-(1-se[2])*(1-se[3]), -se[2]*se[3]); ul3 = min(se[2]*(1-se[3]),(1-se[2])*se[3])a12 = mean(c(ll1,ul1)); a13 = mean(c(ll2,ul2)); a23 = mean(c(ll3,ul3))ll4 = max(-(1-sp[1])*(1-sp[2]), -sp[1]*sp[2]); ul4 = min(sp[1]*(1-sp[2]),(1-sp[1])*sp[2])ll5 = max(-(1-sp[1])*(1-sp[3]), -sp[1]*sp[3]); ul5 = min(sp[1]*(1-sp[3]),(1-sp[1])*sp[3])ll6 = max(-(1-sp[2])*(1-sp[3]), -sp[2]*sp[3]); ul6 = min(sp[2]*(1-sp[3]),(1-sp[2])*sp[3])b12 = mean(c(ll4,ul4)); b13 = mean(c(ll5,ul5)); b23 = mean(c(ll6,ul6))ll71 = -(se[1]*se[2]*se[3]+se[1]*a23+se[2]*a13+se[3]*a12)ll72 = -((1-se[1])*(1-se[2])*se[3]-(1-se[1])*a23-(1-se[2])*a13+se[3]*a12)ll73 = -((1-se[1])*se[2]*(1-se[3])-(1-se[1])*a23+se[2]*a13-(1-se[3])*a12)ll74 = -(se[1]*(1-se[2])*(1-se[3])+se[1]*a23-(1-se[2])*a13-(1-se[3])*a12)ll7 = max(ll71, ll72, ll73, ll74)ul71 = (1-se[1])*se[2]*se[3]+(1-se[1])*a23-se[2]*a13-se[3]*a12ul72 = se[1]*(1-se[2])*se[3]-se[1]*a23+(1-se[2])*a13-se[3]*a12ul73 = se[1]*se[2]*(1-se[3])-se[1]*a23-se[2]*a13+(1-se[3])*a12ul74 = (1-se[1])*(1-se[2])*(1-se[3])+(1-se[1])*a23+(1-se[2])*a13+(1-se[3])*a12ul7 = min(ul71, ul72, ul73, ul74)a123 = mean(c(ll7,ul7))ll81 = -(sp[1]*sp[2]*sp[3]+sp[1]*b23+sp[2]*b13+sp[3]*b12)ll82 = -((1-sp[1])*(1-sp[2])*sp[3]-(1-sp[1])*b23-(1-sp[2])*b13+sp[3]*b12)ll83 = -((1-sp[1])*sp[2]*(1-sp[3])-(1-sp[1])*b23+sp[2]*b13-(1-sp[3])*b12)ll84 = -(sp[1]*(1-sp[2])*(1-sp[3])+sp[1]*b23-(1-sp[2])*b13-(1-sp[3])*b12)ll8 = max(ll81, ll82, ll83, ll84)ul81 = (1-sp[1])*sp[2]*sp[3]+(1-sp[1])*b23-sp[2]*b13-sp[3]*b12ul82 = sp[1]*(1-sp[2])*sp[3]-sp[1]*b23+(1-sp[2])*b13-sp[3]*b12ul83 = sp[1]*sp[2]*(1-sp[3])-sp[1]*b23-sp[2]*b13+(1-sp[3])*b12ul84 = (1-sp[1])*(1-sp[2])*(1-sp[3])+(1-sp[1])*b23+(1-sp[2])*b13+(1-sp[3])*b12ul8 = min(ul81, ul82, ul83, ul84)b123 = mean(c(ll8,ul8))print(paste("list(pr=0.5, se=c(", se[1], ",", se[2], ",", se[3], "), sp=c(", sp[1], ",", sp[2], ",", sp[3], "), a12=", a12, ",a13=", a13, ",a23=", a23, ",b12=", b12, ",b13=", b13, ",b23=", b23, ",a123=", a123, ",b123=", b123, ", r2=c(51,1,2,5,1,6,9,995))"), quote=F)###333pr =0.5; se=c(0.98,0.9, 0.98) ; sp= c(0.999,0.999,0.999);p=array (0, 8)ll1 = max(-(1-se[1])*(1-se[2]), -se[1]*se[2]); ul1 = min(se[1]*(1-se[2]),(1-se[1])*se[2])ll2 = max(-(1-se[1])*(1-se[3]), -se[1]*se[3]); ul2 = min(se[1]*(1-se[3]),(1-se[1])*se[3])ll3 = max(-(1-se[2])*(1-se[3]), -se[2]*se[3]); ul3 = min(se[2]*(1-se[3]),(1-se[2])*se[3])a12 = mean(c(ll1,ul1)); a13 = mean(c(ll2,ul2)); a23 = mean(c(ll3,ul3))ll4 = max(-(1-sp[1])*(1-sp[2]), -sp[1]*sp[2]); ul4 = min(sp[1]*(1-sp[2]),(1-sp[1])*sp[2])ll5 = max(-(1-sp[1])*(1-sp[3]), -sp[1]*sp[3]); ul5 = min(sp[1]*(1-sp[3]),(1-sp[1])*sp[3])ll6 = max(-(1-sp[2])*(1-sp[3]), -sp[2]*sp[3]); ul6 = min(sp[2]*(1-sp[3]),(1-sp[2])*sp[3])b12 = mean(c(ll4,ul4)); b13 = mean(c(ll5,ul5)); b23 = mean(c(ll6,ul6))ll71 = -(se[1]*se[2]*se[3]+se[1]*a23+se[2]*a13+se[3]*a12)ll72 = -((1-se[1])*(1-se[2])*se[3]-(1-se[1])*a23-(1-se[2])*a13+se[3]*a12)ll73 = -((1-se[1])*se[2]*(1-se[3])-(1-se[1])*a23+se[2]*a13-(1-se[3])*a12)ll74 = -(se[1]*(1-se[2])*(1-se[3])+se[1]*a23-(1-se[2])*a13-(1-se[3])*a12)ll7 = max(ll71, ll72, ll73, ll74)ul71 = (1-se[1])*se[2]*se[3]+(1-se[1])*a23-se[2]*a13-se[3]*a12ul72 = se[1]*(1-se[2])*se[3]-se[1]*a23+(1-se[2])*a13-se[3]*a12ul73 = se[1]*se[2]*(1-se[3])-se[1]*a23-se[2]*a13+(1-se[3])*a12ul74 = (1-se[1])*(1-se[2])*(1-se[3])+(1-se[1])*a23+(1-se[2])*a13+(1-se[3])*a12ul7 = min(ul71, ul72, ul73, ul74)a123 = mean(c(ll7,ul7))ll81 = -(sp[1]*sp[2]*sp[3]+sp[1]*b23+sp[2]*b13+sp[3]*b12)ll82 = -((1-sp[1])*(1-sp[2])*sp[3]-(1-sp[1])*b23-(1-sp[2])*b13+sp[3]*b12)ll83 = -((1-sp[1])*sp[2]*(1-sp[3])-(1-sp[1])*b23+sp[2]*b13-(1-sp[3])*b12)ll84 = -(sp[1]*(1-sp[2])*(1-sp[3])+sp[1]*b23-(1-sp[2])*b13-(1-sp[3])*b12)ll8 = max(ll81, ll82, ll83, ll84)ul81 = (1-sp[1])*sp[2]*sp[3]+(1-sp[1])*b23-sp[2]*b13-sp[3]*b12ul82 = sp[1]*(1-sp[2])*sp[3]-sp[1]*b23+(1-sp[2])*b13-sp[3]*b12ul83 = sp[1]*sp[2]*(1-sp[3])-sp[1]*b23-sp[2]*b13+(1-sp[3])*b12ul84 = (1-sp[1])*(1-sp[2])*(1-sp[3])+(1-sp[1])*b23+(1-sp[2])*b13+(1-sp[3])*b12ul8 = min(ul81, ul82, ul83, ul84)b123 = mean(c(ll8,ul8))print(paste("list(pr=0.5, se=c(", se[1], ",", se[2], ",", se[3], "), sp=c(", sp[1], ",", sp[2], ",", sp[3], "), a12=", a12, ",a13=", a13, ",a23=", a23, ",b12=", b12, ",b13=", b13, ",b23=", b23, ",a123=", a123, ",b123=", b123, ", r2=c(1,1,2,5,1,6,9,995))"), quote=F)
