# Supplementary material for: Bayesian evaluation of three serological tests for the diagnosis of bovine brucellosis in Bangladesh
Source: Epidemiol Infect. 2019 Jan 25;147:e73. doi: 10.1017/S0950268818003503 (PMC6518595; doi:10.1017/S0950268818003503)
Supplement: Supplementary file 1 [file S0950268818003503sup001.zip › S2_STARD-BLCM_checklist.docx]

|  | **Section & Topic** | **No** | **Item** | **Reported on page #** |
| --- | --- | --- | --- | --- |
|  |  |  |  |  |
|  | **TITLE OR ABSTRACT** |  |  |  |
|  |  | **1** | **Bayesian evaluation of three serological tests for the diagnosis of bovine brucellosis in Bangladesh** | 01 |
|  | **ABSTRACT** |  |  |  |
|  |  | **2** | Structured summary of study design, methods, results, and conclusions  (for specific guidance, see STARD for Abstracts) | 02 |
|  | **INTRODUCTION** |  |  |  |
|  |  | **3** | Scientific and clinical background, including the intended use and clinical role of the **tests under evaluation** | 04 |
|  |  | **4** | Study objectives and hypotheses, **this study was conducted to estimate the Se and Sp of three serological tests, iELISA, RBT and SAT using Bayesian latent class models** | 04 |
|  | **METHODS** |  |  |  |
|  | *Study design* | **5** | Cross-sectional | 05 |
|  | *Participants* | **6** | Eligibility criteria **and description of the source population** | 06 |
|  |  | **7** | On what basis potentially eligible participants were identified  (such as symptoms, results from previous tests, inclusion in registry): randomly from one district out of 64 and one government dairy farm in Bangladesh | 05 & 06 |
|  |  | **8** | Where and when potentially eligible participants were identified (setting, location and dates): In Mymensingh district and central cattle breeding and dairy farm during September 2007 to August 2008. | 05 |
|  |  | **9** | Whether participants formed a consecutive, random or convenience series: Random | 05 & 06 |
|  | *Test methods* | **10** | **Description of the tests under evaluation**, in sufficient detail to allow replication, **and/or cite references: Reference cited** | 07 |
|  |  | **11** | Rationale for choosing the **tests under evaluation in relation to their purpose: Availability, cheap, rapidity and easy to use.** | 06 |
|  |  | **12** | Definition of and rationale for test positivity cut-offs or result categories of **the tests under evaluation**, distinguishing pre-specified from exploratory: one qualitative and two quantitative with already known result categories and cut-offs. | 07 |
|  |  | **13** | Whether clinical information was available to the performers or readers of **the tests under evaluation: no** |  |
|  | *Analysis* | **14a** | **BLCM model** for estimating measures of diagnostic accuracy | 08-09 |
|  |  | **14b** | **Definition and rationale of prior information and sensitivity analysis** | 10-11 |
|  |  | **15** | How indeterminate results **of the tests under evaluation** were handled: **N/A** |  |
|  |  | **16** | How missing data **of the tests under evaluation** were handled: **No missing data** |  |
|  |  | **17** | Any analyses of variability in diagnostic accuracy, distinguishing pre-specified from exploratory | 11 |
|  |  | **18** | Intended sample size and how it was determined | 05-06 |
|  | **RESULTS** |  |  |  |
|  | *Participants* | **19** | Flow of participants, using a diagram: **N/A, cross-sectional survey and testing in parallel** |  |
|  |  | **20** | Baseline demographic and clinical characteristics of participants | 12 |
|  |  | **21** | **Not applicable: the distribution of the targeted conditions is unknown, hence the use of BLCM** |  |
|  |  | **22** | Time interval and any clinical interventions between **the tests under evaluation: N/A** |  |
|  | *Test results* | **23** | Cross tabulation of the **tests’ results (or for continuous tests results their distribution by infection stage): Provided** | 24 |
|  |  | **24** | Estimates of diagnostic accuracy **under alternative prior specification** and their precision (such as 95% **credible/probability intervals**) | 27 |
|  |  | **25** | Any adverse events from performing **the tests under evaluation** |  |
|  | **DISCUSSION** |  |  |  |
|  |  | **26** | Study limitations, including sources of potential bias, statistical uncertainty, and generalisability | 16 |
|  |  | **27** | Implications for practice, including the intended use and clinical role of **the tests under evaluation in relevant settings (clinical, research, surveillance etc.)** | 17 |
|  | **OTHER INFORMATION** |  |  |  |
|  |  | **28** | Registration number and name of registry |  |
|  |  | **29** | Where the full study protocol can be accessed |  |
|  |  | **30** | Sources of funding and other support; role of funders | Belgian Development Cooperation; The funders had no role in study design, data collection and  analysis, decision to publish, or preparation of the manuscript |
|  |  |  |  |  |

STARD - BLCM

STARD-BLCM stands for “Standards for the Reporting of Diagnostic accuracy studies that use Bayesian Latent Class Models” and is a modification of the STARD statement (which was recently updated to STARD2015). STARD-BLCM aims to facilitate improved quality of reporting for diagnostic accuracy studies that use Bayesian latent class models in the absence of a reference standard. The proposed modifications are relevant to both Bayesian and frequentist estimation methods but the focus is on the former.

More information for STARD (STARD2015) can be found at: [http://www.equator-network.org/reporting-guidelines/stard](http://www.equator-network.org/reporting-guidelines/stard/)

More information for STARD-BLCM can be found at: [http://www.equator-network.org/reporting-guidelines/stard-blcm](http://www.equator-network.org/reporting-guidelines/stard-blcm/)
