# Supplementary material for: Bayesian evaluation of three serological tests for the diagnosis of bovine brucellosis in Bangladesh
Source: Epidemiol Infect. 2019 Jan 25;147:e73. doi: 10.1017/S0950268818003503 (PMC6518595; doi:10.1017/S0950268818003503)
Supplement: Supplementary file 1 [file S0950268818003503sup001.zip › S3_Meta-analysis_of_diagnostic_tests.docx]

**Meta-analysis for the selection of prior information on sensitivity and specificity of iELISA, RBT and SAT: Rahman et al (2018) Bayesian evaluation of three serological tests for the diagnosis of bovine brucellosis in Bangladesh.**

Based on a review of the literature, no information was available regarding the true prevalence and test sensitivities and specificities for bovine brucellosis in Bangladesh. Therefore, prior information from other similar studies was used. Based on several studies obtained from the literature, a meta-analysis (Random effect) was performed using “metandi” in Stata 12.1 [1]. To perform meta-analysis using “metandi”, the number of true positives (TP), true negatives (TN), false positives (FP) and false negatives (FN) were to be known for each study. We included those studies published until 2012 which had information on TP, FP, TN, and FN. Tables 1, 2 and 3 respectively showed data for iELISA, RBT and SAT which were used for meta-analysis.

Table 1. Indirect ELISA data for the meta-analysis

| Source | Se (95% CI) | Sp (95% CI) | TP | FP | FN | TN | Cut-off value |
| --- | --- | --- | --- | --- | --- | --- | --- |
| Van Aert et al. [2] (S 19) | 71.4 (41.9, 91.6) | 100 (99.5, 100)* | 10 | 0 | 4 | 677 | 3× OD of the blank |
| Van Aert et al. [2] (S 2308) | 70.6 (44.0, 89.7) | 100 (99.5, 100)* | 12 | 0 | 5 | 677 | 3× OD of the blank |
| Dohoo et al. [3] | 96.6 (92.6, 98.7) | 99.0 (98.3, 99.5) | 168 | 11 | 6 | 1117 | OD ≥ 0.22 |
| Dohoo et al. [3] | 94.8 (90.4, 97.6) | 99.5 (98.8, 99.8) | 165 | 6 | 9 | 1122 | OD ≥ 0.26 |
| Dohoo et al. [3] | 94.3 (89.7, 97.2) | 99.8 (99.4, 99.9) | 164 | 2 | 10 | 1126 | OD ≥ 0.30 |
| Dohoo et al. [3] | 92.5 (87.5, 96.0) | 99.9 (99.5, 99.9) | 161 | 1 | 13 | 1127 | OD ≥ 0.34 |
| Saegerman et al. [4] | 100 (69.2, 100)* | 97.1 (95.8, 98.1) | 10 | 27 | 0 | 909 | OD ≥ 0.25 (2.5 IU / ml) |
| Abernethy et al. [5] | 67.2 (59.5, 74.4) | 100 (99.9, 100)* | 109 | 0 | 53 | 2663 | 𝛼 |
| Uzal et al. [6] | 98.9 (96.1, 99.9) | 98.8 (96.4, 99.7) | 182 | 3 | 2 | 240 | 31% of positive serum |
| Samartino et al. [7] | 98.2 (97.2, 98.9) | 98.6 (97.1, 99.4) | 982 | 7 | 18 | 493 | 40% of positive serum |

𝛼 = 100 x (OD450 value of the sample − OD450 value of the negative control)/(mean OD450 value of the positive control − OD450 value of the negative control); *97.5% CI; Se=Sensitivity; Sp= Specificity CI= Confidence Interval; TP= True Positive; FP= False Positive; TN= True Negative; FN= False Negative

Table 2. Rose Bengal Test (RBT) data for the meta-analysis

| Source | Se (95% CI) | Sp (95% CI) | TP | FP | FN | TN |
| --- | --- | --- | --- | --- | --- | --- |
| Van Aert et al. [2] (S 19) | 35.7 (12.8, 64.9) | 100 (99.5, 100)* | 5 | 0 | 9 | 677 |
| Van Aert et al. [2] (S 2308) | 76.5 (50.1, 93.2) | 100 (99.5, 100)* | 13 | 0 | 4 | 677 |
| Abernethy et al. [5] | 67.2 (59.5, 74.4) | 100 (99.9, 100)* | 104 | 0 | 58 | 2663 |
| Samartino et al. [7] | 98.2 (97.2, 98.9) | 98.6 (97.1, 99.4) | 961 | 11 | 39 | 489 |
| Dajer et al. [8] | 96.2 (94.1, 97.7) | 68.8 (64.5, 72.9) | 481 | 153 | 9 | 337 |
| Muma et al. [9] | 92.6 (85.4, 96.9) | 80.0 (63.1, 91.6) | 88 | 7 | 7 | 28 |
| Mainer Jaime et al. [10] | 100 (98.1, 100)* | 86.4 (79.1, 91.9) | 189 | 17 | 0 | 108 |

*97.5% CI; Se=Sensitivity; Sp= Specificity CI= Confidence Interval; TP= True Positive; FP= False Positive; TN= True Negative; FN= False Negative

Table 3. Slow Agglutination Test (SAT) data for the meta-analysis

| Source | Se (95% CI) | Sp (95% CI) | TP | FP | FN | TN | Cut-off value |
| --- | --- | --- | --- | --- | --- | --- | --- |
| Van Aert et al. [2] | 35.7 (12.8, 64.9) | 100 (99.5, 100)* | 4 | 0 | 10 | 677 | 30 IU/ ml |
| Lord et al. [11] | 100 (93.6, 100)* | 100 (96.4, 100)* | 56 | 0 | 0 | 100 | 100 IU/ ml |
| Abernethy et al. [5] | 75.9 (68.6, 82.3) | 98.6 (98.0, 98.9) | 123 | 38 | 39 | 2624 | 31 IU / ml |
| Stemshorn et al. [12] | 68.9 (61.2, 75.8) | 99.5 (98.6, 99.8) | 115 | 4 | 52 | 726 | 60 IU / ml |

*97.5% CI; Se=Sensitivity; Sp= Specificity CI= Confidence Interval; TP= True Positive; FP= False Positive; TN= True Negative; FN= False Negative

References

1. Harbord, RM and Whiting, P (2009) metandi: Meta-analysis of diagnostic accuracy using hierarchical logistic regression. *Stata Journal* **9**, 211.
2. Van Aert, A et al. (1984) A comparative study of ELISA and other methods for the detection of *Brucella* antibodies in bovine sera. *Veretrinary Microbiology* 10, 13-21.
3. Dohoo, IR et al. (1986) A comparison of five serological tests for bovine brucellosis. *Canadian Journal of Veterinary Research* **50**, 485-493.
4. Saegerman, C et al. (2004) Evaluation of the serum i-ELISA using monoclonal antibodies and protein G as peroxidase conjugate for the diagnosis of bovine brucellosis. *Veterinary Microbiology* **100**, 91-105.
5. Abertnethy, DA et al. (2012) Field trail of six serological tests for bovine brucellosis. *The Veterinary Journal* **191**, 364-370.
6. Uzal FA, Carrasco AE, Nielsen K, Echaide S and Cabrera RF, (1996) An indirect ELISA using a monoclonal anti *IgG*1 enzyme conjugate for the diagnosis of bovine brucellosis. *Veterinary Microbiology* **52**, 175-180.
7. Samartino L, Gregoret R, Gall D and Nielsen K, (1999) Fluorescence polarization assay: Application to the diagnosis of bovine brucellosis in Argentina. *Journal of Immunoassay* **20**, 115-126.
8. Dajer, A et al. (1999) Evaluation of a fluorescence-polarization assay for the diagnosis of bovine brucellosis in M´exico. *Preventive Veterinary Medicine* **40**, 67-73.
9. Muma JB, Samui KL, Oloya J, Munyeme M and Skjerve E, (2007) Risk factors for brucellosis in indigenous cattle reared in livestock-wildlife interface areas of Zambia. *Preventive Veterinary Medicine* **80**, 306-317.
10. Mainar-jaime, RC et al. (2005) Specificity dependence between serological tests for diagnosing bovine brucellosis in *Brucella*-free farms showing false positive serological reactions due to *Yersinia enterocolitica* O:9. *Canadian Veterinary Journal* **46**, 913-916.
11. Lord VR, Rolo MR and Cherwonogrodzky JW, (1989) Evaluation of humoral immunity to *Brucella* spp. in cattle by use of an agar-gel immunodiffusion test containing a polysaccharide antigen. *American Journal of Veterinary Research* **11**, 1813- 1816.
12. Stemshorn, BW et al. (1985) A comparison of standard serological tests for the diagnosis of bovine brucellosis in Canada. *Canadian Journal of Comparative Medicine* **49**, 391-394.
